# Editorials

**Published:** 1898-10

**Authors:** 


### EDITORIAL.

### WHERE IN 1899?

1899 will be an important era in the veterinary world. The Seventh International Congress in Baden-Baden will be a mecca for the learned and progressive devotees of our profession in August, and the work of the American Veterinary Medical Association following in September should awaken us one and all to the importance of earnest work and our duties in association circles.

The place of meeting for 1899 is an all-important matter to be decided, and every thought should be directed as to where the best interests of our association may be conserved.

By general consent it turns to the east in 1899, and Washington, New York, and Boston have all been named for our convening.

The Capital City of our land is always an attractive point, and no member of our association who has not been privileged to see the seat of popular national government, the city of magnificent distances, but will want to embrace the opportunity; and all of those who have looked upon its fair features and attractions will yearn to see it again in its added beauty and ever-changing character of public life, with its many departments of public usefulness, its wonderful collections of relics and curiosities, its boundless storehouses of knowledge; its matchless galleries of paintings and, not the least, its close association with the work of our profession through the Bureau of Animal Industry; the veterinary schools of the nation's capital seat and the many members of our association and of the profession. Its railroad facilities are all that can be desired, and its hotel accommodations such as to afford every choice of elegance and price.

Greater New York in all its acquired power and territory, cosmopolitan in every sense, with every kind and varied attraction, offers much for our consideration. The centre of the commercial life of all Americas, its facilities for receiving and entertaining are boundless and unlimited. Its geographical location favors the entire East, and the facilities of reaching the first city of our land leave none without advantages in this direction. It affords the most prolific sources and means for the study of every aspect of our professional work. Its colleges, private veterinary hospitals, bacteriological laboratories, its medical colleges, its museums, its dairy interests, its health bureau, its car stables, its corporation stables, its riding schools, its complete boarding and munificent private stables—every type and kind of horse in every kind of work are there to be seen and studied. The city's parks, museums, galleries, gardens, places of amusement are there to afford enjoyment, pleasure, and profit to all. Her rivers and bay, her Coney Island and near-by seaside resorts make it a wonderfully attractive place for a convention city.

The New York State Society and Greater New York's county organization are there to aid the work. The alumni centre of two of the oldest of our American veterinary colleges, with the largest number of active veterinarians in private practice, all enjoying the boundless opportunities of such a great city; its recent entertainment and holding of one of the most successful meetings ever held by the State Society; for many years the place of holding the semi-annual meetings of the U. S. V. M. A., and that it would be as far east as perhaps some of our Western members could well attend, are all potent reasons of strength for New York as our mecca in 1899.

And Boston, the seat of learning, with its hallowed institutions of knowledge that have done so much for the growth and development of our whole land; for twenty-five years the centre of attraction for our annual meeting; the trysting place of a large percentage of those oldest in membership and activity in our association's history; the centre of great movements in the study and control of our animal plagues; its educational work in the creation of veterinary sanitary police systems for her many cities, towns, and boroughs; for many years the centre of one of the strongest State associations in the land, and which would still feel the valued impulse of our presence among them for still grander and better work; rapidly becoming a centre of alumni associations of the various veterinary colleges of North America; without a visit from the parent organization since 1892, and with vivid memories in everyone's mind of that meeting who was privileged to enjoy that happy occasion; with her great seat of learning—Harvard—and her department of veterinary science; her kindred institutions of learning; her wonderful laboratories of scientific investigation; her abattoirs, and every facility for the promotion of scientific thought and action; with a large number of our members in the New England States, and where we should be adding many more; amid environments of land and sea, to attract and benefit all who might be favored by attendance, Boston should be a strong, active seeker for the convention of 1899. Washington, New York, and Boston. Where in 1899?

---

THE editorial staff of the JOURNAL sincerely appreciates the many expressions given by the members of the profession all over the land in timely and forcible denials and just support of our editor, Dr. R. S. Huidekoper, as Chief Surgeon of the First Army Corps of Volunteers. The endeavor by base calumnies, distorted reports, efforts of others to shirk just responsibility and place the same on the shoulders of Lieut.-Col. Huidekoper, will be thrown back upon his ignorant and false accusers in due time, and were it possible to punish the yellow journals, who are only curs of journalism, this might well be done. These many unsolicited acts of warm friendship by members of the profession are truly significant of the common bond that binds us together.

---

NOW that our government is spending millions of dollars among our own people for products of the farm, ranche, and loom, let it inaugurate at once a postal savings institution through which the people may safely invest their savings, and let there be no more foreign bond-issues, but our interest remain at home among the people who have made our country what it is by their thrift and industry.

---

PENNSYLVANIA'S semi-annual meeting at Pittsburg, in September, was one of the most enjoyable in the Association's history. The entertainment by the local committee was extremely pleasant and thoroughly enjoyed by all who were privileged to attend. The luncheon given was generous to the extreme. The banquet tendered by City Veterinarians McNeil and Lacock, respectively of Pittsburg and Allegheny, was spread at Hotel Henry in an exceptionally attractive way, and the menu choice in selection and elegantly served. The special entertainment tendered by our colleague, Veterinarian McNeil, of Pittsburg, by night, was of such a nature as to be long remembered by young and old. All in all, it was a royal reception, a feast of good things of mind and matter, and marks another bright spot in Association history of the Keystone State.

